# Fungal–bacterial interactions in the respiratory tract: from microbial interplay to clinical implications

**DOI:** 10.1128/mbio.02330-25

**Published:** 2026-09-15

**Authors:** Lindsay G. Pauls, Matheus Mertz Ribeiro, Gustavo H. Goldman, Stephen K. Dolan

**Affiliations:** 1 Department of Genetics and Biochemistry, Eukaryotic Pathogens Innovation Center, Clemson University 215869, Clemson, South Carolina, USA; 2 Faculdade de Ciências Farmacêuticas de Ribeirão Preto, Universidade de São Paulo 28133, São Paulo, Brazil; 3 National Institute of Science and Technology in Human Pathogenic Fungi, São Paulo, Brazil; Vallabhbhai Patel Chest Institute, Delhi, India

**Keywords:** *Aspergillus fumigatus*, *Candida albicans*, *Cryptococcus neoformans*, bacterial-fungal interplay, lower respiratory tract, lung microbiome

## Abstract

The respiratory tract harbors complex, dynamic microbial communities during infection, including bacteria (e.g., *Pseudomonas aeruginosa* and *Staphylococcus aureus*), fungi (e.g., *Aspergillus fumigatus* and *Cryptococcus neoformans*), and the resident respiratory microbiota. Within this ecosystem, microbes engage in cooperative and competitive interactions shaped by nutrient availability, immune modulation, and interkingdom chemical signaling, collectively influencing microbial persistence and pathogenic potential. While traditional studies have largely focused on single-pathogen infections, emerging evidence highlights the role of polymicrobial interactions—both synergistic and antagonistic—between pathogens and the respiratory microbiota in shaping disease progression in respiratory disorders. This minireview summarizes recent advances in understanding these complex microbial interactions and highlights key knowledge gaps and future directions in this rapidly evolving field.

## INTRODUCTION

### The respiratory tract as a polymicrobial ecosystem

**Fig 1:**
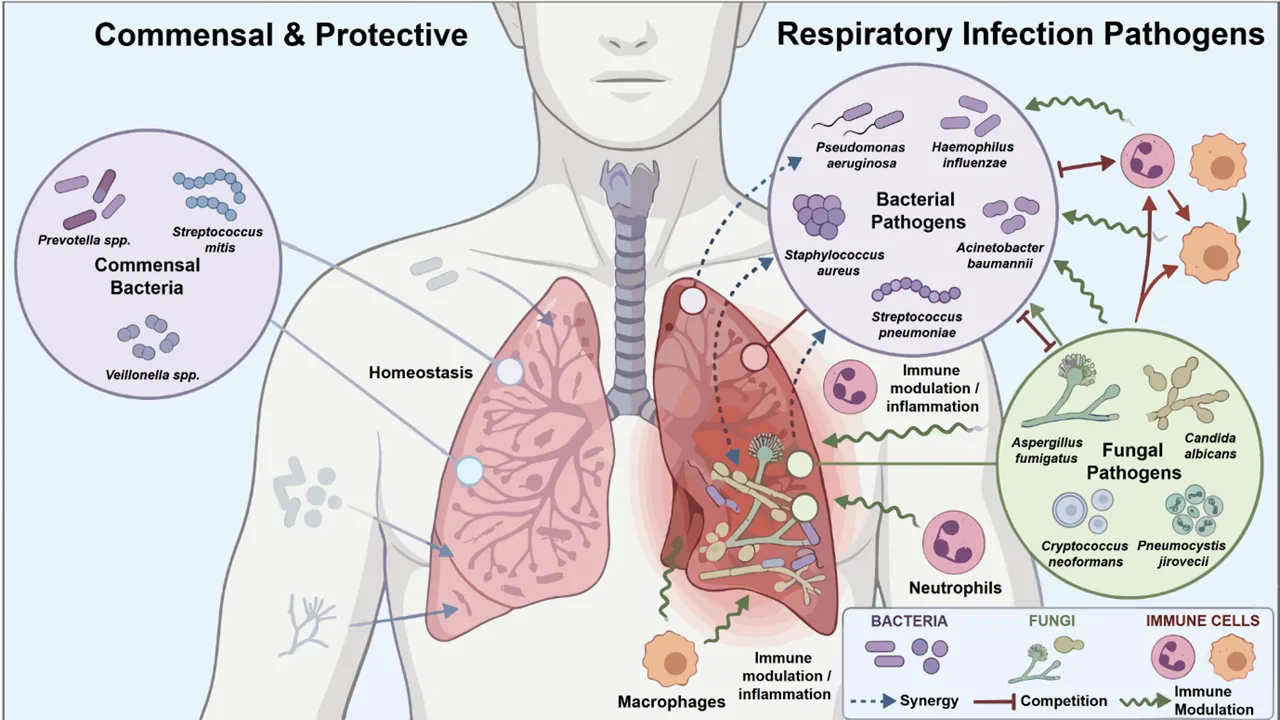
The human respiratory tract contains diverse microbial communities in which commensal and protective microorganisms, pathogens, and host immune cells interact to shape respiratory health and disease. Protective and commensal bacteria (left), including *Prevotella*, *Veillonella*, and *Streptococcus mitis*, contribute to colonization resistance by occupying ecological niches, competing with invading microbes, and promoting immune homeostasis. In contrast, respiratory infection is associated with the expansion of bacterial pathogens such as *Streptococcus pneumoniae*, *Haemophilus influenzae*, *Staphylococcus aureus*, *Pseudomonas aeruginosa*, and *Acinetobacter baumannii*, as well as fungal pathogens including *Aspergillus fumigatus*, *Candida albicans*, *Cryptococcus neoformans*, and *Pneumocystis* species. Interactions among bacteria, fungi, and viruses can be antagonistic (competition) or synergistic, influencing pathogen persistence and disease severity. These microbial interactions are closely linked to host immune responses, as shown by the recruitment and activation of immune cells such as macrophages, which modulate inflammation, pathogen clearance, and tissue damage. Overall, the figure highlights how the balance between commensal microbes, pathogenic organisms, and immune cell activity determines respiratory tract outcomes.

Respiratory infections are among the most common infectious diseases worldwide and range from mild, self-limiting illnesses to severe, life-threatening conditions (Fig. 1). They are broadly classified by anatomical site into upper respiratory tract infections (URTIs) and lower respiratory tract infections (LRTIs) and by epidemiology into community-acquired and hospital-acquired infections (1, 2). Although viruses account for the majority of respiratory infections, bacterial and fungal pathogens are major contributors to severe disease, chronic infection, and poor clinical outcomes, particularly in vulnerable patient populations (3–6). Infections caused by emerging fungal pathogens are of increasing concern, yet their interactions with other respiratory microbes remain understudied. Studies from other anatomical sites can therefore provide a valuable framework for understanding potential respiratory interactions. Although local conditions influence whether and to what extent microbial processes are expressed, microbes may retain the genetic capacity for similar cellular processes and interkingdom interactions across host niches. Mechanistic insights from other body sites can thus generate testable hypotheses about fungal–bacterial interactions in the respiratory tract.

### Upper and lower respiratory tract infections

URTIs affect the nasal passages, sinuses, pharynx, and larynx and include conditions such as the common cold, sinusitis, and pharyngitis (7). These infections are predominantly viral; however, bacterial pathogens including *Streptococcus pyogenes*, *Streptococcus pneumoniae*, *Haemophilus influenzae*, and *Moraxella catarrhalis* can cause primary disease or secondary infections (8–10). Fungal involvement in URTIs is uncommon and typically restricted to immunocompromised hosts, where organisms such as *Candida* and *Aspergillus* species may colonize or invade the upper airway and sinuses (11–13). Fungal balls, a non-invasive form of fungal rhinosinusitis, can develop in the sinuses of immunocompetent individuals, with *Aspergillus* species acting as the leading cause (14–16). High-throughput sequencing has revealed fungal–bacterial community associations that may contribute to the pathogenesis of sinus fungal balls (17, 18).

LRTIs involve the trachea, bronchi, and lungs and include bronchitis and pneumonia. They represent a leading cause of hospitalization and mortality worldwide (19). Common bacterial causes of LRTIs include *S. pneumoniae*, *Staphylococcus aureus*, *H. influenzae*, *Mycoplasma pneumoniae*, and *Legionella pneumophila* (20). Fungal pathogens are increasingly recognized as important causes of LRTIs, particularly in immunocompromised individuals, with *Aspergillus fumigatus*, *Candida albicans*, *Cryptococcus neoformans*, and *Pneumocystis jirovecii* among the most clinically significant (5, 21). Immunocompetent individuals can develop fungal LRTIs, with *Coccidioides* and *Histoplasma* serving as the most common causes of mycoses endemic to the Americas (5, 22). Increasing cases of invasive pulmonary aspergillosis and chronic pulmonary aspergillosis (CPA) have been detected in individuals with chronic obstructive pulmonary disease (COPD) who are otherwise immunocompetent (23, 24). Recent studies of fungal balls in lung cavities of individuals with CPA have reframed them as complex polymicrobial biofilms, and multi-omics approaches have revealed metabolic exchanges between multiple fungal and bacterial species (18, 25, 26).

### Hospital-acquired infections

Hospital-acquired respiratory infections, including ventilator-associated pneumonia, are frequently caused by antibiotic-resistant bacteria such as *Pseudomonas aeruginosa*, *Acinetobacter baumannii*, and methicillin-resistant *S. aureus* (27). Opportunistic fungi, particularly *Aspergillus* and *Candida* species, are also common in hospitalized and critically ill patients, where immunosuppression, invasive procedures, and antibiotic use disrupt normal respiratory microbiota (6, 21). Antimicrobial exposure and loss of microbial diversity can reduce colonization resistance by eliminating competitors, altering nutrient availability, and disrupting microbial–immune homeostasis, thereby creating ecological opportunities for *Aspergillus* and *Candida* species (6, 28, 29).

### High-risk populations

Certain populations experience a disproportionate burden of severe and chronic respiratory infections. People with cystic fibrosis (CF) exhibit impaired mucociliary clearance, promoting persistent colonization by bacterial pathogens such as *P. aeruginosa* and *S. aureus* (30–32). Fungi, particularly *A. fumigatus*, are increasingly recognized as key contributors to CF lung disease, causing allergic, chronic, or invasive infections and engaging in complex interactions with bacterial pathogens that influence disease progression and treatment response (33–35). In addition to being found in other respiratory conditions such as COPD and acute exacerbations of COPD, *Candida* species are also frequently detected in CF respiratory samples; however, they are generally considered colonizers rather than primary drivers of pulmonary disease, though their presence may influence airway microbial ecology and host immune responses (36–41).

Immunocompromised individuals, including patients with malignancies, HIV/AIDS, or those receiving immunosuppressive therapies, are highly susceptible to opportunistic respiratory infections (2, 8). In these patients, fungi such as *Aspergillus*, *Cryptococcus*, and *Pneumocystis* are major causes of pneumonia, often occurring alongside bacterial infections. Diagnosis and management are complicated by overlapping clinical and radiographic presentations, reduced sensitivity of conventional diagnostic methods, and the need to balance empiric antimicrobial therapy against drug toxicity, interactions, and the patient’s underlying immunosuppression (42–44). *Candida* species are commonly isolated from respiratory specimens in this population, but true *Candida* pneumonia remains rare and typically reflects disseminated disease or profound immunosuppression rather than primary pulmonary invasion (45, 46).

Transplant recipients, particularly lung and hematopoietic stem cell transplant patients, are at high risk for both bacterial and fungal respiratory infections due to prolonged immunosuppression and healthcare exposure (47, 48). Early post-transplant infections are often bacterial and hospital-acquired, whereas later infections frequently involve opportunistic fungi, with *Aspergillus* species being a leading cause of invasive pulmonary disease (49). *Candida* species are important causes of early post-transplant invasive disease, most often manifesting as bloodstream or disseminated infections, with pulmonary involvement occurring infrequently and usually as part of systemic candidiasis rather than isolated pneumonia (45, 50).

Beyond direct infection, fungi also contribute to immune-mediated respiratory disease, with allergic bronchopulmonary aspergillosis (ABPA) representing the best-characterized example (6, 51, 52). *C. albicans* is reported to be the major causative agent of allergic bronchopulmonary mycosis (ABPM), which is less common than ABPA and where disease is caused by fungi other than *Aspergillus* species (6, 53). In addition, severe asthma with fungal sensitization and allergic fungal rhinosinusitis highlight a broader spectrum of diseases in which fungal antigens drive chronic airway inflammation in the absence of tissue invasion (54, 55). Together, these conditions underscore the capacity of airway-associated fungi to shape respiratory disease through immune dysregulation rather than direct pathogenicity.

### Why fungi matter

There has been a growing recognition that fungi are not merely passive colonizers or rare opportunistic pathogens but active participants in the respiratory microbial ecosystem, where dynamic, multi-kingdom microbial populations are shaped by nutrient availability, environmental exposures, antimicrobial therapies, and the interactions between microbes and the host immune system (6, 28, 29, 56–58). Advances in culture-independent diagnostics have revealed a far greater diversity and prevalence of airway fungi than previously appreciated, particularly in chronic lung disease and immunocompromised states (59, 60). At the same time, widespread antibiotic use, increasing numbers of immunosuppressed patients, and longer survival of individuals with chronic respiratory conditions have created ecological niches that favor fungal persistence and fungal–bacterial interactions (61). These interactions can alter microbial virulence, antimicrobial tolerance, and host immune responses, with important consequences for disease progression and therapeutic efficacy (62). Together, these insights highlight the urgent need to integrate fungi into conceptual models of respiratory infection and inflammation, moving beyond bacteria-centric frameworks to better understand and manage polymicrobial respiratory disease.

## PRINCIPLES OF FUNGAL–BACTERIAL–HOST INTERACTIONS IN THE RESPIRATORY TRACT

Within the respiratory tract, fungi, bacteria, and the host coexist in a spatially and immunologically complex environment where access to nutrients, exposure to antimicrobial stresses, and host immune pressures collectively shape microbial behavior (63). Rather than acting independently, fungal and bacterial pathogens engage in dynamic interactions that influence colonization, persistence, and pathogenicity. These interactions are governed by coordination of shared resources, the production of antagonistic or cooperative chemical signals, physical associations within structured communities, and host immune responses that both constrain and are shaped by microbial activity (64–68).

### Fungal–bacterial interactions in other host niches inform respiratory disease

**Fig 2:**
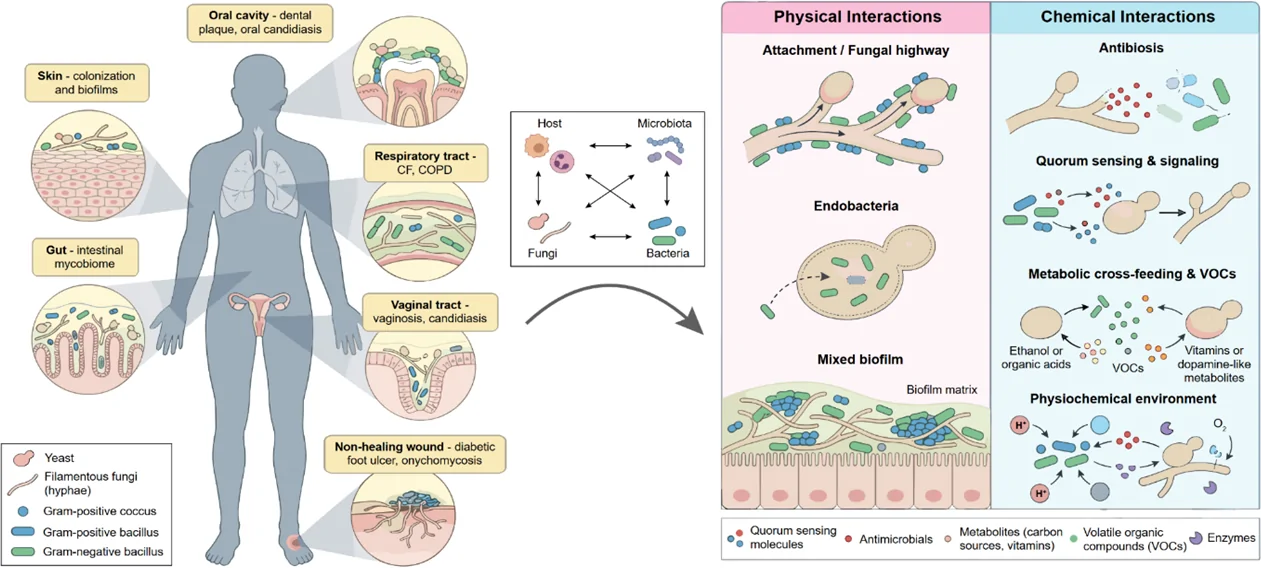
Multi-site fungal–bacterial interactions in the human host and their underlying physical and chemical mechanisms. Schematic overview of the major anatomical niches in which fungi coexist with bacteria in the human body (left) and the principal modes of interaction operating at the microscale (right). Fungal–bacterial communities are depicted across diverse host-associated environments, including the skin (colonization and mixed-species biofilms), oral cavity (dental plaque and oral candidiasis), gastrointestinal tract (intestinal mycobiome), respiratory tract (e.g., cystic fibrosis and chronic obstructive pulmonary disease), vaginal tract (vaginosis and vulvovaginal candidiasis), and chronic wounds such as diabetic foot ulcers and onychomycosis. Representative fungal morphotypes and bacterial taxa are illustrated to emphasize the polymicrobial nature of these niches. The right panel highlights key physical and chemical interaction modalities that shape fungal–bacterial community structure and function. Physical interactions include direct attachment of bacteria to fungal cells or hyphae (the “fungal highway”), intracellular residence of bacteria within fungal cells (endobacteria), and the formation of mixed-species biofilms embedded in a shared extracellular matrix. Chemical interactions include antibiosis via secretion of antimicrobial compounds, interkingdom quorum sensing and signaling, metabolic cross-feeding mediated by diffusible metabolites and volatile organic compounds (VOCs), and reciprocal modification of the physiochemical environment (e.g., pH, oxygen tension, and redox state). Together, these interactions influence microbial fitness, spatial organization, virulence, antimicrobial tolerance, and host outcomes across mucosal and cutaneous ecosystems.

Until relatively recently, healthy lungs were believed to be a sterile environment, and microbial presence was attributed to disease states (69, 70). Due to the developing nature of this field of study, much of what is currently understood about fungal–bacterial interactions during infection has emerged from studies in non-respiratory host niches, including the intestine, oral cavity, skin, wounds, and central nervous system (71–76). In parallel, interactions studied in terrestrial environments such as soil and aquatic systems have also been major contributors to our understanding of mechanistic interactions (77–79). These environments have provided tractable models for dissecting interkingdom competition, cooperation, and immune modulation, yielding conceptual frameworks that are increasingly applicable to the respiratory tract (Fig. 2). Insights gained from these systems highlight conserved interaction strategies that transcend anatomical location and inform how similar dynamics may operate in the airway.

The intestinal microbiota has been particularly informative in revealing how fungal–bacterial interactions shape immune homeostasis and disease susceptibility. Interactions between *Candida* species and bacterial communities in the gut influence fungal colonization, bacterial community structure, and host immune education, including Th17 and regulatory T cell–mediated responses (80, 81). Disruption of bacterial populations through antibiotics can promote fungal expansion, while fungal metabolites and cell wall components can, in turn, modulate bacterial behavior and host inflammation. These findings provide a conceptual parallel to the respiratory tract, where antibiotic exposure and immune dysregulation similarly precede fungal persistence and disease.

Conversely, antagonistic interactions between *C. albicans* and *P. aeruginosa* have been extensively studied in wound and burn infections, where bacterial secondary metabolites suppress fungal filamentation, while fungal presence alters bacterial virulence and host inflammatory responses (82–85). These niche-dependent outcomes underscore how local environmental conditions shape the balance between cooperation and competition—an idea directly relevant to the heterogeneous microenvironments of the infected airway.

Studies of *A. fumigatus* in wound and burn models have demonstrated how bacterial competitors may influence fungal growth and immune activation, where increased invasive hyphal growth was associated with decreased function of both macrophages and neutrophils (86, 87). Similarly, research on *C. neoformans* in the central nervous system (CNS) has revealed how interactions with resident microbes and immune cells affect fungal persistence and dissemination to the CNS following pulmonary infection (88, 89). Although these niches differ markedly from the lung, they highlight conserved strategies of immune modulation and stress adaptation that are likely relevant during pulmonary colonization and infection.

Collectively, studies from diverse host niches demonstrate that fungal–bacterial interactions are context-dependent but mechanistically conserved, involving shared principles of metabolic competition, chemical signaling, physical association, and immune modulation. Applying these lessons from the intestine and other non-respiratory environments to the airway provides a powerful framework for understanding polymicrobial respiratory disease and highlights the value of cross-niche perspectives in advancing respiratory mycology.

### Metabolic competition and cross-feeding

Competition for limiting nutrients is a fundamental driver of fungal–bacterial interactions in the respiratory tract. Iron is a particularly critical resource, with both bacteria and fungi deploying high-affinity acquisition systems to overcome host-imposed nutritional immunity (66, 90, 91). In addition to iron, competition for carbon, nitrogen, and trace metals further shapes community composition, especially in mucus-rich environments such as the cystic fibrosis airway (64, 90, 92, 93).

In contrast to purely competitive interactions, metabolic cross-feeding can promote microbial coexistence. Fungal metabolism can generate fermentation products, organic acids, or oxygen gradients that are exploited by bacterial partners, while bacterial enzymatic activity can liberate nutrients, such as carbon and nitrogen sources, that support fungal growth (94–97). These cooperative interactions may enhance community stability and persistence, particularly under chronic infection conditions, while simultaneously altering host inflammatory responses.

### Microbial chemical warfare

Fungal–bacterial interactions are frequently mediated by the production of bioactive secondary metabolites that function as chemical weapons or signaling molecules. Bacteria such as *P. aeruginosa* secrete phenazines, quinolones, and rhamnolipids that inhibit fungal growth, disrupt fungal biofilms, or modulate fungal morphogenesis (98, 99). Conversely, fungi produce metabolites, including gliotoxin and other mycotoxins, which can impair bacterial growth or interfere with host immune cells (100, 101).

Importantly, many of these compounds exert context-dependent effects, functioning as antagonistic molecules at high concentrations but acting as signaling cues at lower levels (99, 102). Chemical interactions can therefore reshape microbial behavior without necessarily eliminating competitors, promoting adaptive responses that influence virulence, stress tolerance, and antimicrobial resistance.

### Physical interactions and biofilm architecture

Physical associations between fungi and bacteria further influence infections by shaping biofilm structure and function. Fungal hyphae can serve as scaffolds for bacterial attachment, facilitating spatial organization and protection from environmental stresses (68). Additionally, endosymbiotic relationships between fungi and endofungal bacteria can result in increased fungal virulence and altered morphology (103, 104). Bacteria even exploit specialized fungal survival structures such as chlamydospores, entering and persisting within these fungal tissues (105). Mixed-species biofilms often exhibit increased resistance to antimicrobial agents and host immune clearance compared to single-species communities (66, 106, 107).

In chronic airway diseases, structured biofilms provide microenvironments with steep gradients in oxygen, nutrients, and pH, fostering microbial heterogeneity and phenotypic diversity (108, 109). These spatial features can enhance microbial persistence while complicating treatment, as pathogens embedded within biofilms may exhibit reduced susceptibility to both antifungal and antibacterial therapies (63, 110).

### Host immunity as an interaction mediator

Host immune responses play a central role in shaping fungal–bacterial interactions in the respiratory tract. Innate immune mechanisms, including epithelial barrier function, mucus production, and the activity of neutrophils and macrophages, impose selective pressures that influence microbial community composition (90, 111, 112). Neutrophil-derived calprotectin, which is abundant in the lung during inflammation, has been recently shown to induce immune-mediated β-lactam tolerance in *S. aureus* through zinc sequestration, thereby limiting antibiotic-driven bacterial clearance (113)

Fungal–bacterial interactions can synergistically amplify or suppress inflammation, altering disease severity and progression. *C. albicans* and *S. aureus* have been shown to synergistically dysregulate the host immune response by increasing proinflammatory cytokines (114, 115). In chronic infection settings, sustained immune activation can drive tissue damage that further reshapes the airway environment, reinforcing microbial dysbiosis and promoting long-term colonization (116).

## FUNGI AS DRIVERS OF POLYMICROBIAL DYNAMICS IN THE RESPIRATORY TRACT

The respiratory tract microbiome, containing relatively low biomass compared to other anatomical sites, is dominated by bacterial communities, with fungal burden less characterized (28, 59, 70, 116–118). Despite this, fungi exert a disproportionate influence on airway ecology through their metabolic activity, physical structure, and capacity to modulate host immunity. Distinct fungal pathogens engage in unique interactions with bacterial communities and host defenses, shaping polymicrobial dynamics in organism-specific ways (6, 59, 116, 117). Examining these interactions through an organism-centered lens highlights both shared principles and pathogen-specific strategies that drive respiratory disease.

### Aspergillus fumigatus

*A. fumigatus* is the most extensively studied fungal pathogen in the respiratory tract and a central model for understanding fungal–bacterial interactions. In chronic lung diseases such as cystic fibrosis and in immunocompromised hosts, *A. fumigatus* coexists with diverse bacterial communities, often alongside dominant bacterial pathogens. Its metabolic flexibility, filamentous growth, and production of secondary metabolites enable effective competition for nutrients and occupation of protected niches within the airway (60, 119, 120).

Growing evidence demonstrates that *A. fumigatus* engages in extensive and mechanistically diverse interactions with bacteria and host-associated microbiota, shaping microbial community structure and pathogen fitness. At the molecular level, *A. fumigatus* deploys a range of secondary metabolites and secreted factors that mediate both cooperative and antagonistic interactions with bacterial neighbors. The production of isocyanide metabolites from the *xan* biosynthetic gene cluster exemplifies this versatility, as these chalkophore-like compounds contribute to fungal copper homeostasis while exerting copper-dependent antimicrobial activity against competing microbes, revealing a dual role in metal acquisition and intermicrobial competition (121, 122). Similarly, *A. fumigatus* secretes the antibacterial endopeptidase CwhA, which directly cleaves bacterial peptidoglycan and lyses gram-positive bacteria during coculture; the inhibition of neighboring bacteria leads to enhanced fungal fitness due to the reduced competition and antagonistic interactions (123). This provides clear evidence that pathogenic fungi deploy enzymatic weapons analogous to bacterial autolysins to antagonize bacterial competitors. Additionally, the *Streptomyces rapamycinicus-*produced antimicrobial azalomycin F acts as an interkingdom signaling molecule that activates the transcriptional regulator SmpR in *A. fumigatus*. This results in the induction of the fumicycline biosynthetic gene cluster (BGC) along with several other antibacterial secondary metabolite BGCs (124).

In polymicrobial communities, *A. fumigatus* also engages in reciprocal chemical interactions that reshape metabolism and stress responses in both kingdoms. Bacterial metabolites can profoundly alter fungal physiology, as illustrated by exposure to *Klebsiella pneumoniae* culture filtrates, which suppress fungal growth while simultaneously inducing gliotoxin biosynthesis, potentially amplifying fungal immunomodulatory capacity despite reduced biomass (125, 126). Interactions with *P. aeruginosa* are particularly well characterized and reveal both antagonistic and cooperative dimensions. *P. aeruginosa* secretes dirhamnolipids that inhibit fungal β−1,3-glucan synthase, driving compensatory cell wall remodeling and reduced echinocandin susceptibility, thereby linking bacterial metabolites directly to antifungal drug tolerance (98). In parallel, the *P. aeruginosa* quorum-sensing molecule PQS modulates *A. fumigatus* iron metabolism in an iron-dependent manner, inhibiting growth under iron limitation while paradoxically promoting fungal biofilm formation and development under iron-replete conditions (99). These findings underscore how environmental context, particularly metal availability, determines whether bacterial–fungal interactions are competitive or synergistic.

Beyond diffusible metabolites, physical and matrix-mediated interactions further reinforce cross-kingdom associations. The fungal exopolysaccharide galactosaminogalactan (GAG) promotes cooperative biofilm formation with *P. aeruginosa*, facilitating bacterial adherence to fungal hyphae and enhancing bacterial tolerance to colistin through incorporation of GAG into the biofilm matrix (127). Complementing this, *A. fumigatus* secondary metabolism directly influences polymicrobial biofilm ecology; pyripyropene A is required for robust dual biofilm formation with *P. aeruginosa*, modulates host macrophage responses, and contributes to virulence *in vivo*, identifying fungal secondary metabolites as central regulators of interspecies biofilm architecture and pathogenicity (128). At the same time, bacteria have evolved specific defenses against fungal toxins, exemplified by the induction of the dithiol oxidase DnoP in *P. aeruginosa*, which detoxifies gliotoxin via a mechanism convergent with the fungal enzyme GliT, highlighting conserved evolutionary pressures imposed by fungal natural products (101).

These molecular and cellular interactions scale to broader ecological and host-associated consequences. *In vivo*, respiratory infection with *A. fumigatus* disrupts the lung microbiota, creating microaerophilic niches that favor enrichment of anaerobic bacteria and altering distal gut microbiota and host metabolism, implicating the gut–lung axis in fungal infection–associated dysbiosis (129). Clinical studies further support the relevance of such interactions, as fungal ball rhinosinusitis is associated with cooccurrence of *A. fumigatus* and *Haemophilus influenzae*, suggesting structured bacterial–fungal consortia rather than fungal monocultures in disease lesions (17, 130, 131). Antibiotic perturbation of these microbial communities can have profound immunological consequences: microbiota disruption impairs neutrophil reactive oxygen species (ROS)-dependent killing of *A. fumigatus*, increasing susceptibility to invasive infection and demonstrating that bacterial communities indirectly support antifungal host defense (132). Moreover, subinhibitory antibiotic exposure itself can act as a signaling cue, reshaping bacterial and fungal metabolomes and altering interkingdom chemical exchange, as shown for trimethoprim-induced activation of the *ham* gene cluster in *Burkholderia cenocepacia* and downstream effects on *Aspergillus* siderophore and sterol metabolism (133).

Collectively, these studies establish *A. fumigatus* as an active participant in complex bacterial and microbiota-associated networks, engaging in chemical warfare, metabolic cooperation, physical association, and immune modulation. This emphasizes the need to consider polymicrobial and microbiota contexts when studying aspergillosis pathogenesis and developing therapeutic strategies.

### Candida albicans

#### A model for fungal–bacterial interaction biology beyond classical pneumonia

Although traditionally associated with mucosal and systemic infections, *Candida albicans* is frequently detected in respiratory samples, particularly in critically ill and immunocompromised patients. While often regarded as a colonizer rather than a primary pulmonary pathogen, *C. albicans* can influence airway microbial ecology through its metabolic activity, morphogenetic flexibility, and immunomodulatory properties. Members of the genus *Candida*, particularly *C. albicans*, function as dynamic participants in polymicrobial communities, engaging in bidirectional interactions with bacteria that shape microbial ecology, antimicrobial susceptibility, and host disease outcomes. These interactions are mediated through a combination of chemical signaling, metabolic exchange, physical association, and immune modulation and can promote either antagonism or cooperative pathogenicity depending on environmental and host context (134–136).

At the molecular level, *Candida* actively exchanges metabolites and signals with bacterial neighbors that reprogram virulence and stress responses in both kingdoms. Classic antagonistic interactions with *P. aeruginosa* are governed by conditional metabolic cues: fungal ethanol production induces phosphate starvation responses in *P. aeruginosa*, driving phenazine biosynthesis and antifungal activity in a phosphate-dependent manner (137). Conversely, fungal competition for essential micronutrients such as magnesium can remodel bacterial envelope physiology and reduce bacterial susceptibility to polymyxin antibiotics, highlighting nutrient sequestration as a key axis of interkingdom competition (138). Bacteria also exploit fungal quorum signals; in the gut, *Yersinia pseudotuberculosis* senses the fungal autoinducer tyrosol to activate an antifungal type VI secretion system (T6SS), directly suppressing *Candida* colonization *in vivo* (139). In addition, *A. baumannii* can use a T6SS to directly intoxicate and kill *Candida* via dedicated antifungal effectors (140). These findings build on earlier work demonstrating that bacterial T6SSs can deploy dedicated antifungal effectors with distinct cellular targets, establishing direct fungal intoxication as a conserved bacterial strategy (141).

In other settings, *Candida*–bacterial interactions instead promote cooperative virulence through metabolic cross-feeding. In the gut, *C. albicans* enhances *Salmonella Typhimurium* pathogenicity by releasing extracellular arginine following direct bacterial–fungal contact and effector delivery, which amplifies *Salmonella* type III secretion, invasion, and systemic dissemination (142). Similar metabolic coupling underlies synergistic infections with *S. aureus*, where fungal ribose catabolism controlled by the transcriptional regulator Zcf13 removes ribose-mediated repression of the staphylococcal *agr* quorum-sensing system, enabling toxin-driven lethality during intra-abdominal coinfection (143). Copper availability can further stabilize cooperative interactions, as proposed for *C. albicans*–*S. aureus* biofilms in which asymmetric copper handling supports enhanced dual-species biomass and persistence (144). Gause and Johnson (95) define a metabolically driven interkingdom interaction between *C. albicans* and *Enterococcus faecalis*, demonstrating how reciprocal nutrient exchange supports coexistence. Using *in vivo* models, they uncovered that fungal citrate production fuels bacterial growth, while bacterial metabolism generates formate, which *C. albicans* detoxifies via induced formate dehydrogenases. This work highlights how cross-feeding and metabolic byproducts shape cooperative yet conditional interactions between bacteria and fungi (95). Although studied in the gut, the findings provide a conceptual framework for understanding how similar metabolic exchanges may influence microbial persistence in respiratory infections. Together, these studies highlight how fungal metabolism can directly tune bacterial quorum sensing, toxin production, and virulence through shared nutrient pools.

Physical association and spatial organization are equally central to *Candida*–bacterial ecology. Fungal hyphae act as structural scaffolds that promote bacterial attachment, dispersal, and coexistence in complex environments. In oral biofilms, *C. albicans* forms interkingdom assemblages with *Streptococcus mutans* that exhibit emergent group-level mobility, enhanced stress tolerance, and increased cariogenic potential, reframing disease-promoting biofilms as multicellular fungal–bacterial collectives (107). Hyphal architecture also determines competitive outcomes in confined microenvironments, where directional fungal growth enables stable coexistence and reproducible spatial patterning with bacterial pathogens that would otherwise dominate on open surfaces (145). Beyond serving as passive scaffolds, hyphae can intensify bacterial–bacterial antagonism by increasing contact frequency and enabling contact-dependent killing during surface-associated growth, illustrating how fungal morphology can indirectly restructure bacterial competition networks (146).

These interkingdom interactions are further shaped by fungal morphogenetic programs and toxins. Contrary to the view that hyphal growth is incompatible with commensalism, candidalysin—a hyphal-specific toxin—promotes *C. albicans* gut colonization in hosts with intact bacterial microbiota by enhancing early competitive fitness against bacteria, while adaptive immunity later constrains hyphal persistence (147). This dual role underscores the tension between microbial competition and host immune selection in defining commensal versus pathogenic states. In parallel, bacterial partners can modulate fungal drug susceptibility in clinically relevant ways: *P. aeruginosa* increases *C. albicans* sensitivity to amphotericin B in dual-species biofilms through phenazine-mediated mitochondrial stress (82) and synergizes with fluconazole in polymicrobial infection models, partially via bacterial iron sequestration (148). *Escherichia coli* releases metabolites that induce cell wall remodeling in *C. albicans*, resulting in increased sensitivity to amphotericin B and increased resistance to fluconazole and caspofungin (149). Conversely, fungal presence can diminish antibiotic efficacy against bacteria, as seen in *Streptococcus agalactiae*–*Candida* coinfections where polymicrobial growth reduces clindamycin protection and enhances virulence *in vivo* (150).

At the community and host levels, *Candida* interactions with the microbiota profoundly influence disease susceptibility and progression. Pre-colonization with *Candida glabrata* exacerbates *Clostridioides difficile* infection in mice, demonstrating that fungal commensals can prime bacterial pathogenesis and alter enteric disease trajectories (151). In the vaginal tract, *C. albicans* acts as a fungal pathobiont that promotes *S. agalactiae* persistence, ascension, and pathogenicity through a combination of physical association and metabolic cross-talk, emphasizing the importance of interkingdom interactions in mucosal niches (152). Together, these studies argue that *Candida* species are not passive components of the mycobiota but active contributors whose interactions with bacteria can recalibrate virulence, antimicrobial responses, and host immunity.

Collectively, this body of work reframes *Candida* biology within a polymicrobial and microbiota-aware context, in which fungal morphology, metabolism, and signaling integrate with bacterial competition strategies and host responses. Understanding these interkingdom interactions is essential for predicting infection outcomes and designing therapies that account for the microbial community, rather than targeting *Candida* in isolation.

### Cryptococcus neoformans

#### Environmental training grounds for immune evasion and polymicrobial disease

*C. neoformans* causes severe pneumonia and meningoencephalitis, particularly in immunocompromised individuals, yet its interactions with the respiratory microbiota remain comparatively understudied. Following inhalation, *C. neoformans* encounters resident bacterial communities and host immune defenses that influence fungal survival, immune evasion, and dissemination. Its polysaccharide capsule and capacity to modulate host immune responses suggest that interkingdom interactions may significantly affect disease outcomes (88).

Direct antagonistic interactions between *Cryptococcus* and bacteria have been most clearly demonstrated for *P. aeruginosa*. Early work showed that *P. aeruginosa* strongly inhibits the growth of multiple *Cryptococcus* species through secreted, diffusible factors, independent of direct contact (153). Exposure to bacterial supernatants reduced cryptococcal growth, capsule thickness, and melanin production—key virulence determinants—suggesting that bacterial metabolites can directly attenuate fungal pathogenic potential. These findings align with broader observations that *P. aeruginosa* deploys antifungal chemistry against diverse fungi and underscore that *Cryptococcus* is subject to competitive suppression by bacterial neighbors in both environmental reservoirs and host-associated settings.

Beyond chemical antagonism, purely physical interactions may also influence *Cryptococcus*–bacterial ecology. Biophysical analyses reveal that immotile yeast colonies generate thin, dynamic fluid layers at their surface that facilitate bacterial dispersal across otherwise restrictive substrates (154). This mechanism does not rely on active fungal signaling or metabolism, indicating that fungal growth alone can restructure microbial landscapes and promote bacterial spread. Such physical effects may be particularly relevant in soil, pulmonary, or mucosal environments where *Cryptococcus* encounters diverse bacterial communities.

Importantly, bacterial–*Cryptococcus* interactions frequently manifest indirectly through modulation of host immunity rather than direct microbial antagonism. Multiple studies demonstrate that bacterial infection or colonization can prime protective immune responses that enhance resistance to subsequent cryptococcal challenge. In murine models, prior *P. aeruginosa* infection reduces fungal burden and improves survival following *Cryptococcus gattii* infection, an effect associated with enhanced recruitment of neutrophils and inflammatory monocytes and elevated pro-inflammatory cytokine production (155). Similarly, *S. aureus* infection triggers an inflammatory state that confers protection against secondary *C. gattii* infection, limiting fungal dissemination and disease severity through heightened innate immune activation (156). These studies highlight a form of immune conditioning or “trained immunity,” whereby bacterial exposure alters host responsiveness to fungal pathogens.

The complexity of these interactions becomes particularly apparent during simultaneous polymicrobial infection. Cross-kingdom infection of macrophages with bacterial and fungal pathogens induces extensive reprogramming of both host and microbial proteomes, revealing immune- and pathogen-specific adaptive responses that differ markedly from single-pathogen infections (157). In this setting, *C. neoformans* alters its intracellular stress responses, metabolism, and virulence-associated pathways in response to bacterial c-infection, while macrophages mount distinct inflammatory and metabolic programs depending on the microbial combination encountered. These findings reinforce the concept that polymicrobial infections generate emergent host–pathogen states that cannot be inferred from single-organism models.

Clinical and translational perspectives further support the relevance of bacterial–fungal–immune interactions in cryptococcosis. Reviews of pulmonary cryptococcosis emphasize that disease progression reflects a balance between fungal virulence traits, host immune status, and the inflammatory environment of the lung, which is frequently shaped by coinfections, microbiota composition, and underlying pulmonary disease (158). At the pathogen level, proteomic comparisons of *C. neoformans* clinical isolates associated with latent versus lethal infection reveal differential expression of metabolic, stress-response, and immune-evasion pathways (159), suggesting that fungal programs tuned for persistence or immune confrontation may be influenced by microbial competition and immune conditioning *in vivo*.

Collectively, these studies position *Cryptococcus* as a fungal pathogen whose biology and pathogenicity are strongly shaped by interactions with bacteria and host immunity. Bacterial competitors can directly suppress cryptococcal growth or virulence traits, while bacterial infections can indirectly alter susceptibility to cryptococcosis by priming or reprogramming host immune responses. Together with emerging physical and ecological mechanisms, this work argues that cryptococcal disease should be understood within a polymicrobial framework, in which bacterial–fungal interactions and immune context jointly determine infection outcome.

### Emerging and understudied fungal pathogens

In addition to well-characterized fungal pathogens, a growing number of emerging and historically understudied fungi are increasingly implicated in respiratory disease, often in the context of altered host immunity, environmental exposure, or prolonged healthcare-associated risk factors (160–162). Although direct mechanistic studies of fungal–bacterial interactions are limited for many of these organisms, accumulating clinical and ecological evidence suggests that they operate within polymicrobial and immunologically complex airway environments.

*Pneumocystis* species are obligate fungal pathogens that cause pneumonia in immunocompromised hosts, particularly individuals with impaired T cell–mediated immunity. Unlike other respiratory fungi, *Pneumocystis* is particularly difficult to culture, has only recently been successfully cultured *in vitro*, and occupies a highly specialized niche within the alveolar space (163). Despite this restricted lifestyle, *Pneumocystis* infection occurs within a broader microbial and immunological context. *Pneumocystis* species are increasingly recognized not only as opportunistic pathogens but also as active ecological components of the respiratory mycobiome, particularly in early life (164). Longitudinal microbiome studies show that *P. jirovecii* colonization is associated with reproducible shifts in lower airway bacterial community structure during infancy, suggesting that this unculturable fungus may act as a pivotal driver of microbial equilibrium during critical windows of immune and microbiome development (56). These findings challenge the view of *Pneumocystis* as merely a marker of immune dysfunction and instead position it as a contributor to airway ecosystem assembly.

Advances in molecular diagnostics have revealed that *Pneumocystis* carriage is far more common than previously appreciated, complicating the distinction between colonization and disease and underscoring the need for ecological context when interpreting clinical detection (165). At the organismal level, single-cell RNA sequencing has begun to resolve *Pneumocystis* developmental programs, identifying transcriptionally distinct trophic and sexual stages and highlighting sexual reproduction as a central feature of persistence within the lung (166). Together, these studies emphasize *Pneumocystis* as a host-adapted, microbiome-interacting fungus whose biology and clinical relevance extend well beyond classical opportunistic infection paradigms.

Mucorales, including genera such as *Rhizopus* and *Mucor*, have gained heightened attention due to the rise in COVID-19–associated mucormycosis, particularly among patients with diabetes, corticosteroid exposure, or prolonged hospitalization. These infections frequently arise in airways already disrupted by viral infection, bacterial coinfection, and immune dysregulation (167). While specific bacterial–Mucorales interaction mechanisms remain poorly defined, the clinical contexts in which mucormycosis emerges strongly implicate polymicrobial dynamics as a likely contributor to disease progression.

Clinical studies indicate that bacterial coinfections are common in severely ill patients with mucormycosis and may exacerbate tissue damage, complicate diagnosis, and worsen outcomes, yet remain underappreciated in clinical management (168). Mechanistically, work on *Rhizopus microsporus* has revealed that a bacterial endosymbiont is essential for fungal immune evasion and virulence, demonstrating that pathogenicity can emerge from stable fungal–bacterial partnerships rather than fungal traits alone (169). The surge of COVID-19–associated mucormycosis further highlights how immune dysregulation, antibiotic exposure, and hospital-associated microbiota converge to promote severe disease (167), underscoring the need to view mucormycosis through an interkingdom and ecological lens.

*Histoplasma capsulatum* and *Coccidioides* species are traditionally viewed as environmentally acquired, geographically restricted pathogens that cause primary fungal pneumonia following inhalation of spores (170, 171). Increasing recognition of chronic, relapsing, and severe pulmonary disease—particularly in immunocompromised hosts—raises questions about how these fungi interact with resident airway microbiota and bacterial pathogens during infection. Coinfections with bacterial respiratory pathogens have been reported clinically, and immune responses elicited by these fungi may indirectly shape bacterial community structure and airway inflammation (172, 173).

Other emerging fungi are increasingly detected in chronic and critical care settings. Species such as *Scedosporium* and *Exophiala dermatitidis* are frequently co-isolated with bacterial pathogens including *P. aeruginosa* and *S. aureus* in CF airways, while *Trichosporon* species have been reported alongside gram-negative bacteria in ventilated and immunocompromised patients (174–177). These organisms may participate in polymicrobial interactions that influence airway inflammation, microbial persistence, and treatment outcomes.

Together, these emerging and understudied fungal pathogens underscore the need to broaden current models of respiratory infection beyond classical opportunistic fungi. Understanding how environmental and non-canonical fungi integrate into polymicrobial airway ecosystems—particularly under conditions of viral infection, immune suppression, and microbiome disruption—represents an important frontier for future research.

## IMPLICATIONS FOR DIAGNOSIS AND TREATMENT

### Diagnostic challenges

Diagnosing polymicrobial respiratory infections presents substantial challenges. Standard culture-based diagnostics are biased toward fast-growing or easily culturable organisms and often fail to detect fungi or mixed microbial communities. Advances in molecular and culture-independent approaches, including targeted sequencing, metagenomics, and fungal-specific biomarkers, have improved detection of airway fungi and complex microbial communities (60, 178, 179). However, these methods also raise interpretive challenges, particularly in distinguishing colonization from clinically relevant infection. Integrating microbial detection with host immune markers and clinical context will be essential for improving diagnostic accuracy in polymicrobial respiratory disease.

### Therapeutic antagonism and synergy

Fungal–bacterial interactions may influence therapeutic outcomes: antibacterial therapy can remove bacterial competitors and promote fungal overgrowth, while antifungal treatment may reciprocally alter bacterial community structure and virulence. Recent studies indicate that these interactions can also modify treatment efficacy. For example, colistin potentiates echinocandin activity against *A. fumigatus* by increasing membrane permeability and stress sensitivity, whereas many common non-antifungal co-medications antagonize antifungal activity against *C. albicans* by promoting tolerance or resistance, potentially contributing to treatment failure in polymicrobial and clinical settings (180, 181).

Understanding these interactions is particularly important in chronic infections, where prolonged antimicrobial exposure can drive microbial adaptation and resistance. Rational combination therapies that account for interkingdom interactions may improve outcomes but require a deeper understanding of microbial ecology and host responses to avoid unintended consequences.

### Microbiome-aware interventions

Recognizing the respiratory tract as a polymicrobial ecosystem opens new opportunities for microbiome-aware therapeutic strategies. These approaches aim not only to eliminate pathogens but also to modulate microbial communities and host responses to restore respiratory homeostasis. Potential strategies include targeted antimicrobial regimens that minimize collateral damage, interventions that disrupt pathogenic biofilms, and therapies that modulate host immunity to reduce excessive inflammation while preserving microbial control (64, 116).

Although still in early stages, microbiome-informed diagnostics and treatments hold promise for improving management of complex respiratory infections. Integrating ecological principles into clinical practice will be critical for developing more effective, durable, and personalized interventions for polymicrobial respiratory disease.

## OUTSTANDING QUESTIONS AND FUTURE DIRECTIONS

### Defining fungal–bacterial interactions in the respiratory ecosystem

Several respiratory-specific questions remain unresolved. Which fungal–bacterial interactions occur directly within the lung, and how are they shaped by airway nutrients, oxygen gradients, mucus, antimicrobial exposure, and host immunity? Determining whether these interactions affect clinically meaningful outcomes, including lung-function decline, treatment failure, and disease recurrence, will require longitudinal clinical studies integrated with physiologically relevant respiratory models. Understanding microbial behavior within the respiratory tract requires experimental frameworks that move beyond single-pathogen models toward approaches capable of capturing context-dependent fitness, interaction-driven phenotypes, and strain-level diversity. Recent advances in functional genomics, population genomics, and systems biology are transforming how bacterial and fungal pathogens are studied within complex, polymicrobial ecosystems. Together, these technologies provide a foundation for dissecting cooperative and competitive interactions shaped by nutrient availability, antimicrobial exposure, and host-associated environments.

Foundational to this progress are scalable genome-wide perturbation platforms that enable systematic interrogation of gene function. Early advances such as piggyBac transposon mutagenesis in haploid *Candida* species established robust methods to quantify gene essentiality and fitness contributions across conditions, providing an unbiased framework for functional genomics in fungal pathogens (182). These approaches have since been complemented and extended by CRISPR-based technologies, which offer greater precision and flexibility. Notably, hyper-dCas12a–based CRISPR activation and interference (CRISPRa/i) systems now enable inducible, multiplexed gene regulation in *C. albicans* (183), overcoming limitations of lethal knockouts and enabling combinatorial perturbations. Such tools are particularly well suited for probing genetic interactions and regulatory networks that may only emerge under polymicrobial or stress-associated conditions encountered in the respiratory tract.

The power of these platforms is illustrated by recent genome-wide functional landscapes mapping essentiality, drug resistance, and phenotypic diversity across fungal pathogens. In *Cryptococcus neoformans*, Billmyre et al. (184) integrated essentiality profiling with fluconazole response data to disentangle core growth requirements from adaptive resistance mechanisms, highlighting how antifungal tolerance is highly context dependent (184). Similarly, large-scale phenotypic atlases generated through systematic perturbation and multi-dimensional readouts reveal emergent biological features not predictable from sequence alone (185). These phenotype-first approaches provide insight into stress adaptation, virulence-associated traits, and potential interaction interfaces that may be modulated by coinfecting microbes.

Context dependence emerges as a unifying theme across these studies. Pooled CRISPRi screens applied across diverse environments and genetic backgrounds demonstrate that gene essentiality is not static but shaped by both strain variation and environmental inputs (186). This is particularly relevant in the respiratory tract, where nutrient availability, immune pressure, and microbial neighbors fluctuate dynamically. Parallel work in bacterial pathogens reinforces this paradigm: CRISPRi screening in *P. aeruginosa* identified FprB as a synergistic target enhancing gallium-based therapy, underscoring how functional genomics can uncover combination strategies that exploit conditional vulnerabilities rather than single-agent liabilities (187). Adapting such approaches to fungal systems could help identify conditional vulnerabilities and potential combination therapies for polymicrobial infections.

Complementing functional screens, population- and pan-genomic analyses provide the evolutionary and strain-level context necessary to interpret interaction potential. Pan-genome studies of *A. fumigatus* reveal extensive accessory gene diversity and structural variation associated with human infection (188), while high-resolution population analyses demonstrate that recombination drives lineage-specific diversity with functional consequences (189). These findings challenge the assumption of a single “representative” pathogen genotype and suggest that interaction capacity, metabolic flexibility, and drug susceptibility may vary substantially among strains coexisting in the lung. Extending beyond *A. fumigatus*, comparative genomic surveys across clinical *Aspergillus* species further highlight how species- and strain-level variation shapes pathogenicity and antifungal resistance, reinforcing the need for genomics-informed diagnostics and therapeutics (190).

Systems biology approaches are now beginning to bridge genotype, phenotype, and ecological context. Genome-scale metabolic modeling across *A. fumigatus* strains has identified growth dependencies predicted to be modulated by the lung microbiome, suggesting that some metabolic constraints become apparent only when host-associated microbial communities are considered (191). These models provide a quantitative framework for predicting nutrient competition, cross-feeding, and vulnerability nodes that may be exploitable therapeutically. Importantly, such approaches move the field beyond descriptive interaction studies toward mechanistic, testable hypotheses about microbial persistence and pathogenic potential *in vivo*.

At the community level, quantitative frameworks explicitly designed to capture polymicrobial dynamics are beginning to fill a critical gap. Adekoya and Ibberson (2025) introduce experimental and computational tools to measure interaction-driven effects on growth, competition, and drug response, addressing the limitations of single-species infection models. These methods provide a conceptual and technical bridge between reductionist functional genomics and the complex microbial ecosystems present in respiratory disease, enabling systematic exploration of synergistic and antagonistic interactions across kingdoms (192).

Collectively, these advances highlight a shift toward interaction-aware microbiology, in which functional genomics, pan-genomics, and systems modeling are integrated to examine microbial behavior within realistic respiratory contexts. Key priorities include determining which fungal–bacterial interactions occur in the lung, how they vary across respiratory diseases and treatment states, and whether they influence disease progression or therapeutic outcomes. Addressing these questions will require higher-throughput interaction screens, physiologically relevant respiratory models, longitudinal clinical sampling, improved fungal detection, and direct *in vivo* validation. Integrating these approaches will be essential for distinguishing interactions that are mechanistically possible from those that are clinically consequential.

## CONCLUSIONS

### Reframing respiratory fungal infections as polymicrobial diseases

Respiratory fungal infections have traditionally been viewed through a narrow lens focused on invasive disease in immunocompromised hosts. However, accumulating evidence demonstrates that fungi are integral components of polymicrobial respiratory ecosystems, impacting disease through interactions with bacterial pathogens and the host immune system. These interactions influence microbial persistence, immune-mediated pathology, and therapeutic responses across a wide range of respiratory conditions.

Despite the increasing number of cases of fungal respiratory infections and the growing body of research on the fungi responsible, clear clinical conclusions are challenging to draw. In a study of the microbiome of CF sputum samples, it was found that multiple clinical isolates of *C. albicans* developed loss of function mutations in the transcriptional repressor *NRG1* (193). These mutations allowed the *C. albicans* isolates to adopt a virulence-associated filamentous state and resistance to the filamentation-inhibitory effects of molecules released by bacterial pathogens co-isolated from the patient samples. However, although this work demonstrated a clear adaptive fungal phenotype, *C. albicans* relative abundance showed only a weak, albeit statistically significant, negative correlation with percent-predicted forced expiratory volume in 1 s (FEV1% predicted), a measure of lung function. Together, these findings highlight the current gap between mechanistic studies of fungal–bacterial interactions and clinically meaningful outcomes. Addressing this knowledge gap will not only require more investigations of fungal–bacterial interactions within the respiratory tract but a shift from bacteria-focused diagnostics to a polymicrobial perspective. Improved detection of fungal pathogens will be crucial for establishing their interactions with co-colonizing microbes and how these dynamics contribute to respiratory disease progression.

Ultimately, treating respiratory fungal disease as an ecosystem-level problem, rather than a single-pathogen failure, may be essential for overcoming chronic infection, antimicrobial tolerance, and recurrent disease.
